# Binding to m^6^A RNA promotes YTHDF2-mediated phase separation

**DOI:** 10.1007/s13238-019-00660-2

**Published:** 2019-10-22

**Authors:** Jiahua Wang, Liyong Wang, Jianbo Diao, Yujiang Geno Shi, Yang Shi, Honghui Ma, Hongjie Shen

**Affiliations:** 1 Center for Medical Research and Innovation, Shanghai Pudong Hospital, Fudan University Pudong Medical Center and Institutes of Biomedical Sciences, Fudan University, Shanghai, 201399 China; 2 Division of Newborn Medicine and Epigenetics Program, Department of Medicine, Boston Children’s Hospital, Boston, MA 02115 USA; 3 Cell Biology Department, Harvard Medical School, Boston, MA 02115 USA; 4 Key Laboratory of Arrhythmias of the Ministry of Education of China,East Hospital, Tongji University School of Medicine, Shanghai, 200120 China; 5 Division of Endocrinology, Diabetes and Hypertension, Department of Medicine, Brigham and Women’s Hospital, Harvard Medical School, Boston, MA USA

**Dear Editor,**

As one of the most abundant modifications on mRNA in mammal, *N*^6^-methyladenosine (m^6^A) has been demonstrated to play important roles in various biological processes including nuclear RNA export, mRNA splicing, miRNA processing, mRNA degradation and translation (Shi et al., 2019). Importantly, different m^6^A reader proteins have been shown to play central roles in these processes.

YTH (YT521-B homology)-domain containing proteins are members of the conserved m^6^A reader family, which recognize m^6^A via the YTH domain (Hazra et al., 2019). Members of this family have been shown to play a role in mRNA translation and mRNA decay (Wang et al., 2014; Wang et al., 2015; Li et al., 2017; Shi et al.,^2017^). However, mechanisms by which these readers impact various biological processes are still elusive. For instance, YTHDF2 has been shown to regulate mRNA decay and is localized to the membrane-less cytoplasmic P granules (Wang et al., 2014), where mRNA decay occurs. But how YTHDF2 and its associated m^6^A RNAs are localized to this phase-separated granule (Standart and Weil, 2018) remains largely unknown.

As P bodies are liquid-like droplets in cytoplasm (Standart and Weil, 2018), we speculated that YTHDF2 may share liquid-like phase separation (LLPS) features. As with many LLPS proteins, YTHDF2 contains a low complexity (LC) domain (aa 230–383), which includes a glutamine (Q) rich domain (aa 288–383) (Fig. 1A). Interestingly, recombinant YTHDF2 protein containing the LC domain (YTHDF2^aa 230−383^) forms liquid droplets (23 μmol/L protein in 37 mmol/L NaCl, 10% PEG8000), which are sensitive to 1,6-hexanediol (previously shown to specifically disrupt liquid-like assemblies) (Kroschwald et al., 2017) (Fig. 1B), suggesting phase separation. This phase separation ability is dependent on NaCl concentration (Fig. S1A), which is consistent with another report (Ries et al., 2019). Moreover, we found these droplets fuse with each other to form bigger droplets, which further suggests a liquid like phase separation feature of YTHDF2^aa 230−383^ (Fig. S1B, and Video S1). Other members of this family, YTHDF1 and YTHDF3, also contain glutamine (Q) rich domain (Fig. S1C), and also have phase separation ability (Fig. S1D). As YTHDF2 glutamine compositional bias is conserved among vertebrates, to determine whether glutamine richness within the LC domain is important for LLPS of YTHDF2, we changed all glutamine to alanine in YTHDF2^aa 230−383^ (Fig. S1E), and the mutated protein essentially failed to form as many and large LLPS under the same assay condition (Fig. S1F and S1G). Similarly, another phase separation protein MED1 contains conserved serine (S) rich region, and this serine bias is necessary for MED1 phase separation (Sabari et al., 2018). To test whether YTHDF2 forms LLPS in cell, we overexpressed EGFP-YTHDF2 in mouse embryonic stem (mES) cells and human U2OS cells. FRAP assays showed YTHDF2 forms LLPS in both cell lines (Fig. 1C and 1D).

**Figure 1:**
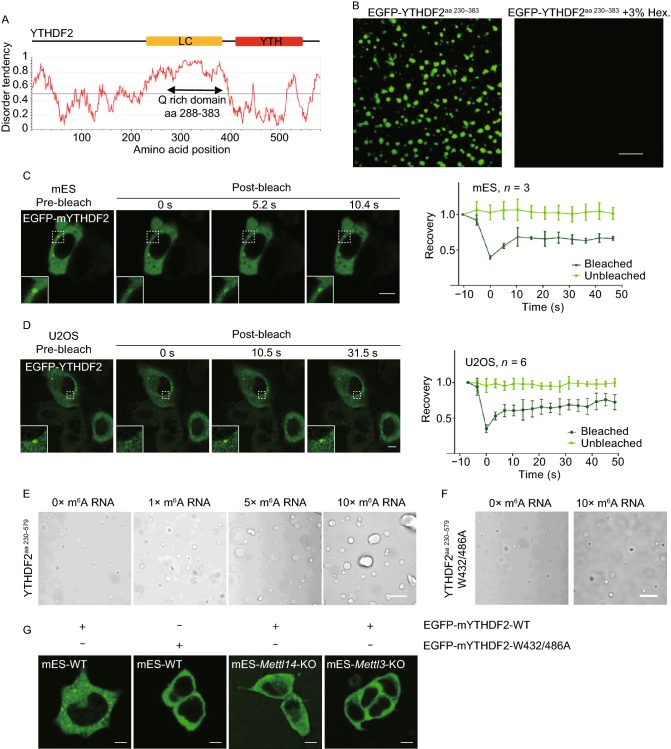
Phase separation of YTHDF2 *in vivo*/*vitro*. (A) Top: Diagram of protein domains of YTHDF2. Bottom: predictions of Intrinsic disorder tendency of YTHDF2 by IUPred2A (https://iupred2a.elte.hu/). Scores above 0.5 indicate disorder. (B) Liquid phase separation of YTHDF2^aa 230−383^ (23 μmol/L YTHDF2, 37 mmol/L NaCl, 10% PEG8000) was sensitive to 1,6-hexanediol (1,6-hex; 3%). Scale bar, 10 μm. (C and D) EGFP-YTHDF2 was exogenously expressed in mES cells (C) or U2OS cells (D). FRAP assays showed YTHDF2 forms LLPS in both cell lines (Left). The line traces represent mean fractional fluorescence (Right). Scale bar, 5 μm (C), 10 μm (D). (E) m^6^A oligos induced liquid like droplet formation of YTHDF2^aa 230−579^ (58 μmol/L YTHDF2, 33 mmol/L NaCl, 17 μmol/L RNA oligos). Scale bar, 10 μm. (F) m^6^A oligos could not induce liquid like droplet formation of W432A/W486A mutated YTHDF2^aa 230−579^ (58 μmol/L YTHDF2, 33 mmol/L NaCl, 17 μmol/L RNA oligos). Scale bar, 10 μm. (G) Wildtype, but not W432A/W486A mutated EGFP-YTHDF2 formed droplets in mESCs. Wildtype EGFP-YTHDF2 failed to form droplets in *Mettl14* or *Mettl3* KO mESCs. Scale bar, 5 μm

Previous studies suggested that RNA plays a crucial role in protein phase separation. For instance, RNA has been shown to facilitate HP1 alpha phase separation on heterochromatin (Strom et al., 2017). Given that YTHDF2 preferentially binds m^6^A RNA (Wang et al., 2014), we hypothesized that m^6^A RNA might promote YTHDF2-mediated phase separation. To test this idea, we constructed a portion of the YTHDF2 protein containing aa 230 to its C terminus (aa 579), which includes both the LC and the YTH domains (Fig. 1A). We identified a condition (13 μmol/L YTHDF2, 37 mmol/L NaCl, 0.74 μmol/L RNA, 10% PEG8000) under which the recombinant YTHDF2^aa 230−579^ protein barely formed LLPS with RNA oligos containing no m^6^A *in vitro* (Fig. S1H, right and S1I). Importantly, however, under the same assay condition, RNA oligos containing one m^6^A modification induced liquid like droplet formation (Fig. S1H, left and S1I). In addition, we found this phase separation enhancement appears to be dependent on the number of m^6^A in the RNA. As shown in Fig. 1E, phase separation mediated by YTHDF2 increases in a manner that is dependent on the number of m^6^A sites in the RNA oligos (50 bp RNAs containing either 0, 1, 5, or 10 m^6^A). These results are consistent with the recent reports that RNA oligos containing multiple m^6^A methylation sites robustly induce droplet formation by YTHDF2 (Gao et al., 2019; Ries et al., 2019). Consistently, induction of phase separation of YTHDF2 by m^6^A RNAs seems to be dependent on the YTH domain, as even the RNA containing 10 m^6^A failed to enhance phase separation in vitro when the m^6^A-binding capability of the YTH domain is compromised (YTHDF2^aa 230−579^ carrying W432A, W486A) (Li et al., 2014) (Fig. 1F). To corroborate this finding, we investigated whether binding of YTHDF2 to m^6^A is necessary for phase separation in vivo by expressing wildtype and the m^6^A-binding defective YTHDF2 (YTH mutated (W432A, W486A)) in mouse ES cells. While wildtype YTHDF2 formed droplets in cell, YTH mutated YTHDF2 did not (Fig. 1G), indicating that the ability of YTHDF2 to bind m^6^A RNA is necessary for YTHDF2 LLPS in vivo. To further confirm this finding, we asked whether YTHDF2 forms LLPS in cells lacking the mRNA m^6^A enzymatic complex, METTL3 or METTL14 (Fig. S1J). We detected no YTHDF2 droplets in these cells (Fig. 1G), further supporting the notion that m^6^A promotes YTHDF2 LLPS in cells.

In summary, we provide both *in vitro* and *in vivo* data demonstrating that m^6^A enhances phase separation of YTHDF2. Although recombinant YTHDF2 itself can phase separate *in vitro*, m^6^A modification significantly enhances this ability, and *in vivo*, YTHDF2 LLPS may in fact be dependent on binding m^6^A mRNAs.

While this manuscript was in preparation, several groups reported that YTHDF family proteins display phase separation potential (Fu and Zhuang, 2019; Gao et al., 2019; Ries et al., 2019) stimulated by m^6^A RNA (Gao et al., 2019; Ries et al., 2019), consistent with our finding that m^6^A promotes the phase separation potential of YTHDF2.

## Electronic supplementary material

Below is the link to the electronic supplementary material.

Supplementary material 1 (DOCX 22 kb)

Supplementary material 2 (MP4 3014 kb)

Supplementary material 3 (DOCX 47 kb)

Supplementary material 4 (PPTX 2065 kb)
