# Supplementary material for: Binding to m6A RNA promotes YTHDF2-mediated phase separation
Source: Protein Cell. 2019 Oct 22;11(4):304–7. doi: 10.1007/s13238-019-00660-2 (PMC7093369; doi:10.1007/s13238-019-00660-2)
Supplement: Supplementary file 1 — Supplementary material 1 (DOCX 22 kb) [file 13238_2019_660_MOESM1_ESM.docx]

**MATERIALS AND METHODS**

**Prediction of Intrinsically Unstructured Proteins**

Predictions of intrinsic disorder tendency of the proteins were accomplished by specifying the accession numbers of the proteins in the "Enter SWISS-PROT/TrEMBL identifier or accession number" field of IUPred2A(Meszaros et al., 2018) ([https://iupred2a.elte.hu/). The](https://iupred2a.elte.hu/).%20The) red line in the result page represents the intrinsic disorder tendency of each protein.

**Cell cultures**

U2OS cells were cultured in Dulbecco’s Modified Eagle’s Medium (DMEM) supplemented with 10% fetal bovine serum (FBS) and 100 U/mL Penicillin/Streptomycin at 37 °C with 5% CO_2_. E14Tg2a murine embryonic stem cells were cultured in DMEM supplemented with 10% FBS, 1% MEM non-essential amino acid, 55 mM β-Mercaptoethanol, 1000 U/mL LIF (Millipore) and 100 U/mL Penicillin/Streptomycin at 37 °C with 5% CO_2_.

**Stable cell lines construction**

EGFP-tagged murine *Ythdf2* was cloned into the pPB-CAG-IRES-Pac plasmid with N-terminal Flag and HA tags. This plasmid was individually co-transfected with pCMV-PBase plasmid into mESCs using Lipofectamine 2000 (Invitrogen) according to the manufacturer’s instruction. Medium was replaced by fresh media with 2 μg/mL Puromycin after 48 hours. After continuous Puromycin selection for 3 days, the survived mESCs were pooled as stable overexpression cell lines.

EGFP-tagged human *YTHDF2* was cloned into the pLenti6.2-V5 plasmid. Lentivirus was made by co-transfection of this vector with VSV-G and psPAX2 in a 3:1:1 ratio into 293T cells. Supernatant at 48 hours post-transfection was collected and concentrated by PEG8000. U2OS cells were seeded in a 6-well plate and infected with lentivirus supernatant in the presence of 5 μg/mL polybrene (Sigma). Medium was replaced by fresh media with 10 μg/mL Blasticidin S at 24 hours post-infection. After continuous Blasticidin S selection for 5 days, survived U2OS cells were pooled as stable infected cell lines.

CRISPR-Cas9 gene targeting was carried out as previously described(Maeder et al., 2013) and the single knockout clones were isolated and then confirmed by Western blot showing undetectable METTL3 and METTL14 protein. Guiding RNA sequences used are: 1) *Mettl3* KO: 5’-GCTTAGGGCCGCTAGAGGTA-3’. 2) *Mettl14* KO: 5’-GTAGCTCAGCAGGTGTGCGG-3’.

**Protein Expression and Purification**

The different truncated fragments of human *YTHDF2* were subcloned into the pMCSG7 vector with the sequence EGFP-GSGS (linker) or not. The *YTHDF2*-LC Q-to-A mutant DNA was synthesized and inserted into the same vector. All proteins were expressed in Rosetta (DE3) cells. Cells in 500 ml LB media were induced overnight at 16 °C with 0.1mM Isopropyl β-D-Thiogalactoside(IPTG) and collected. The pellets were resuspended in 30 ml lysis buffer (50 mM Tris pH7.5, 500 mM NaCl, 10 mM imidazole, 0.01% NP40, 1mM β-Mercaptoethanol) and squeezed three times at 4 °C. After centrifugation at 13000 rpm, 4 °C for 20 minutes, the supernatants were purified with Ni-NTA beads (Smart-Lifesciences) and eluted by elution buffer (50 mM Tris pH7.5, 500 mM NaCl, 200 mM imidazole, 1mM β-Mercaptoethanol). Elutions containing proteins were then analyzed by Coomassie staining and dialyzed against storage buffer (50 mM Tris pH7.5, 100 mM NaCl, 1 mM β-Mercaptoethanol).

**In vitro droplet assay**

Purified proteins were concentrated to the indicated protein concentrationdescribed in the text using Amicon Ultra centrifugal filters (10K MWCO, Millipore) and added to solutions at varying concentrations with the indicated final salt and molecular crowder concentrations. The droplet assay was performed in the following buffer: 50 mM Tris (PH7.5), 1mM β-Mercaptoethanol, and indicated NaCl. The droplet assay was generated by combining YTHDF2 protein or YTHDF2/RNA mixture with 40% PEG8000 ina ratio of 3:1, if PEG8000 was used. The protein solutions were loaded onto a confocal dish and imaged with 10%PEG8000 (Figure 1B, S1A, S1B, S1F, S1H, S1J) or not (Figure 1E, 1F). For detail, in m^6^A-RNA induced droplet assays, probe and protein were firstly mixed in a microtube and imaged on a confocal dish. The sequences of the different m^6^A-RNA probes are 5’-Cy5-CGUGG m^6^ACUGGCU-3’ or 5’-FAM-CGUGGACUGGCU-3’. The sequences of the different sites m^6^A-RNA probes are: 1) 0x m^6^A-RNA: GGACUGGACUGGACUGGACUGGACUGGACUGGACUGGACUGGACUGGACU. 2) 1x m^6^A-RNA: GGACUGGACUGGACUGGACUGGm^6^ACUGGACUGGACUGGACUGGACUGGACU. 3) 5x m^6^A-RNA: GGm^6^ACUGGACUGGm^6^ACUGGACUGGm^6^ACUGGACUGGm^6^ACUGGACUGGm^6^ACUGGACU. 4) 10x m^6^A-RNA: GGm^6^ACUGGm^6^ACUGGm^6^ACUGGm^6^ACUGGm^6^ACUGGm^6^ACUGGm^6^ACUGGm^6^ACUGGm^6^ACUGGm^6^ACU.

**Super-resolution microscopy**

Stable cell lines were seeded in 35mm confocal dishes and imaged with a STED Nanoscopes leica TCS SP8 STED. The images were post-processed by the Leica LAS AF Lite software.

**Fluorescence recovery after photobleaching (FRAP)**

The FRAP was performed on the STED Nanoscopes leica TCS SP8 STED with 488 nm laser. Bleaching was performed over an approximately 1 μm^2^ region using 100% laser power and images were collected every 3.5 seconds (U2OS) or 5.2 seconds (mESC). Fluorescence intensity was measured using the LAS X software. Background intensity was subtracted and values were reported relative to pre-bleaching time points.

**References:**

Maeder, M.L., Linder, S.J., Cascio, V.M., Fu, Y., Ho, Q.H., and Joung, J.K. (2013). CRISPR RNA-guided activation of endogenous human genes. NAT METHODS *10*, 977-979.

Meszaros, B., Erdos, G., and Dosztanyi, Z. (2018). IUPred2A: context-dependent prediction of protein disorder as a function of redox state and protein binding. NUCLEIC ACIDS RES *46*, W329-W337.
