# Supplementary material for: Binding to m6A RNA promotes YTHDF2-mediated phase separation
Source: Protein Cell. 2019 Oct 22;11(4):304–7. doi: 10.1007/s13238-019-00660-2 (PMC7093369; doi:10.1007/s13238-019-00660-2)
Supplement: Supplementary file 3 — Supplementary material 3 (DOCX 47 kb) [file 13238_2019_660_MOESM3_ESM.docx]

**Supplementary Figure 1**

1. Microscopy images of EGFP-YTHDF2^aa 230-383^ droplets (23 μM YTHDF2, 10% PEG8000) subjected to a NaCl gradient, showing NaCl concentration affects the formation of protein droplets. Scale bar, 10 μm.
2. The fusion of YTHDF2^aa 230-383^ droplets (23 μM YTHDF2, 37 mM NaCl, 10% PEG8000) were imaged by microscopy over 120 seconds. Scale bar, 10 μm.
3. Predictions of Intrinsic disorder tendency of YTHDF1 and YTHDF3 by IUPred2A(https://iupred2a.elte.hu/). Scores above 0.5 indicate disorder.
4. Liquid phase separation of YTHDF1^aa 284-362^ (Glutamine rich domain) and YTHDF3^aa 288-388^ (Glutamine rich domain). Scale bar, 10 μm.
5. Sequence alignment of the wildtype and Q-to-A mutant of the Glutamine (Q) rich domain in YTHDF2^aa 230-383^.
6. Q-to-A mutation compromised liquid phase separation of YTHDF2^aa 230-383^ (11 μM YTHDF2, 37 mM NaCl, 10% PEG8000). Scale bar, 10 μm.
7. Droplet numbers of wildtype and Q-to-A mutated YTHDF2^aa 230-383^ proteins. Data are represented as mean from three replicates. Error bars represent SEM of the number of droplets from three replicates. **P < 0.01, t test.
8. Cy5-m^6^A, but not FAM-A RNA oligos induced YTHDF2^aa 230-579^ liquid like droplet formation (13 μM YTHDF2, 37 mM NaCl, 0.74μM RNA, 10% PEG8000).
9. Droplet numbers of Cy5-m^6^A and FAM-A RNA oligos induced YTHDF2^aa 230-579^ proteins. Data are represented as mean from three replicates. Error bars represent SEM of the number of droplets from three replicates. **P < 0.01, t test.
10. Western blot showing Mettl3 protein levels in WT, *Mettl3* KO and Mettl14 protein levels in WT, *Mettl14* KO cell lines.
