# Supplementary material for: Binding to m6A RNA promotes YTHDF2-mediated phase separation
Source: Protein Cell. 2019 Oct 22;11(4):304–7. doi: 10.1007/s13238-019-00660-2 (PMC7093369; doi:10.1007/s13238-019-00660-2)
Supplement: Supplementary file 4 — Supplementary material 4 (PPTX 2065 kb) [file 13238_2019_660_MOESM4_ESM.pptx]

## Slide 1
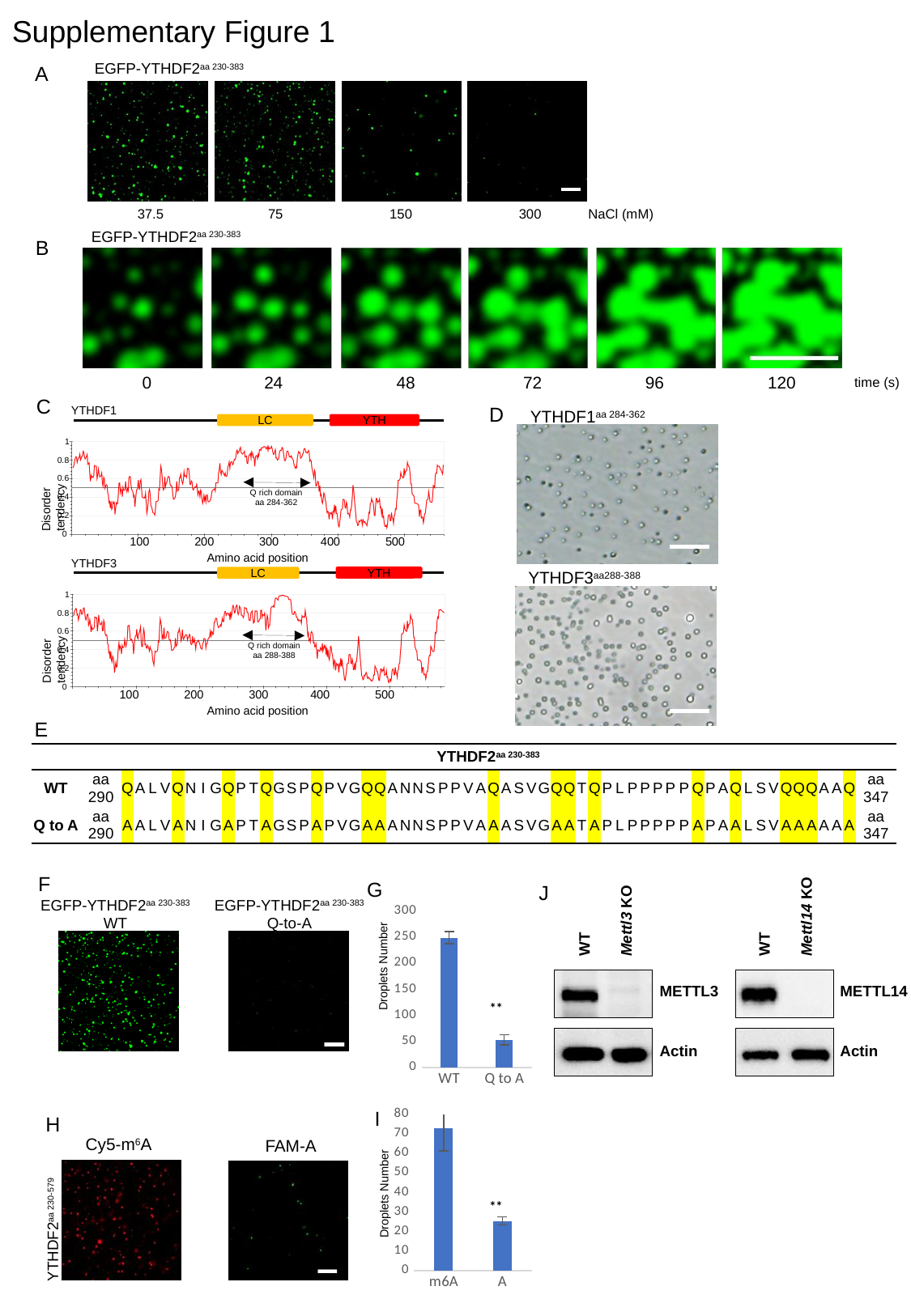

Supplementary Figure 1
EGFP-YTHDF2aa 230-383
NaCl (mM)
37.5
75
150
300
A
EGFP-YTHDF2aa 230-383
B
0
96
24
48
72
120
time (s)
C
D
YTHDF1
LC
YTH
Disorder tendency
 1
0.8
0.6
0.4
0.2
 0
Q rich domain
aa 284-362
100 200 300 400 500
Amino acid position
YTHDF3
YTH
LC
Disorder tendency
 1
0.8
0.6
0.4
0.2
 0
Q rich domain
aa 288-388
100 200 300 400 500
Amino acid position
YTHDF1aa 284-362
YTHDF3aa288-388
E
| | | YTHDF2aa 230-383 | | | | | | | | | | | | | | | | | | | | | | | | | | | | | | | | | | | | | | | | | | | | | | | | | | | | | | | | | | |
| --- | --- | --- | --- | --- | --- | --- | --- | --- | --- | --- | --- | --- | --- | --- | --- | --- | --- | --- | --- | --- | --- | --- | --- | --- | --- | --- | --- | --- | --- | --- | --- | --- | --- | --- | --- | --- | --- | --- | --- | --- | --- | --- | --- | --- | --- | --- | --- | --- | --- | --- | --- | --- | --- | --- | --- | --- | --- | --- | --- | --- |
| WT | aa 290 | Q | A | L | V | Q | N | I | G | Q | P | T | Q | G | S | P | Q | P | V | G | Q | Q | A | N | N | S | P | P | V | A | Q | A | S | V | G | Q | Q | T | Q | P | L | P | P | P | P | P | Q | P | A | Q | L | S | V | Q | Q | Q | A | A | Q | aa 347 |
| Q to A | aa 290 | A | A | L | V | A | N | I | G | A | P | T | A | G | S | P | A | P | V | G | A | A | A | N | N | S | P | P | V | A | A | A | S | V | G | A | A | T | A | P | L | P | P | P | P | P | A | P | A | A | L | S | V | A | A | A | A | A | A | aa 347 |
WT
Mettl14 KO
METTL14
Actin
WT
Mettl3 KO
METTL3
Actin
F
G
J
EGFP-YTHDF2aa 230-383
WT
EGFP-YTHDF2aa 230-383
Q-to-A
### Chart
| Category | |
|---|---|
| WT | 248.66666666666663 |
| Q to A | 53.0 |
Droplets Number
**
I
### Chart
| Category | |
|---|---|
| m6A | 72.66666666666667 |
| A | 25.333333333333325 |H
Cy5-m6A
FAM-A
Droplets Number
**
YTHDF2aa 230-579
